# Confidential Relations between Physician and Patient

**Published:** 1887-04

**Authors:** 


### Editorial.

### CONFIDENTIAL RELATIONS BETWEEN PHYSICIAN AND PATIENT.

We desire to call the special attention of the profession in Georgia to the article in this number of THE JOURNAL "On Certain Medico-Legal Subjects."

There exists much uncertainty amongst physicians in regard to the duties and responsibilities devolving upon them in the practice of their profession, and still greater in regard to the legal privileges which they are entitled to enjoy. Legislative enactments and judicial decisions have failed to remove this uncertainty, even from the legal stand-point. This contribution by a member of the Savannah bar cannot fail to interest our readers. Its publication is well-timed, as it will bring the subject to the consideration of the Medical Association soon to assemble in this city. It would be quite appropriate for that body, if in its wisdom it should so determine, to memorialize the General Assembly upon two points at least suggested in this article—one in relation to expert testimony, which, by previous action of the Association, is already before the Legislature, and the other and more important one of placing the seal of secrecy upon all communications made by patients to their physicians and guarding its sanctity, under all circumstances, by penal enactments, and, as in the case of attorneys, by forbidding courts to extort or admit such communications as evidence in the trial of civil or criminal causes. Upon this point Mr. Abrams appends this advice, which we beg leave to emphasize:

I would advise you, therefore, to ask the present Legislature to place among the class of privileged communications those between physician and patient, in the language of the statutes of New York, which is as follows: "No person duly authorized to practice physic or surgery shall be allowed to disclose any information which he may have acquired in attending any patient in a professional character, and which information was necessary to enable him to prescribe for the patient as a physician, or do any act for him as a surgeon, whether such information be disclosed by the patient or found by the physician or surgeon on examination," and this might also be added to the penal laws and punished as a misdemeanor, the penalty being fine or imprisonment, or both, at the discretion of the court.

